# Development and Organization of the Evolutionarily Conserved Three-Layered Olfactory Cortex

**DOI:** 10.1523/ENEURO.0193-16.2016

**Published:** 2017-01-27

**Authors:** Esther Klingler

**Affiliations:** Department of Basic Neuroscience, University of Geneva, 1211 Geneva 4, Switzerland

**Keywords:** cell identity, cortical layers, migration, neocortex, neurogenesis, olfactory cortex

## Abstract

The olfactory cortex is part of the mammalian cerebral cortex together with the neocortex and the hippocampus. It receives direct input from the olfactory bulbs and participates in odor discrimination, association, and learning (Bekkers and Suzuki, 2013). It is thought to be an evolutionarily conserved paleocortex, which shares common characteristics with the three-layered general cortex of reptiles (Aboitiz et al., 2002). The olfactory cortex has been studied as a “simple model” to address sensory processing, though little is known about its precise cell origin, diversity, and identity. While the development and the cellular diversity of the six-layered neocortex are increasingly understood, the olfactory cortex remains poorly documented in these aspects. Here is a review of current knowledge of the development and organization of the olfactory cortex, keeping the analogy with those of the neocortex. The comparison of olfactory cortex and neocortex will allow the opening of evolutionary perspectives on cortical development.

## Significance Statement

The olfactory cortex is an evolutionarily conserved paleocortex implicated in odor processing. While several studies addressed how the olfactory cortex encodes and processes odorant information, little is known about its precise cellular origin, diversity, and identity. Unraveling where the cells are born and how they migrate toward and settle the olfactory cortex during development is of main importance in understanding its circuit organization and function. In addition, comparing the development of the olfactory cortex with that of the neocortex will help in identifying common evolutionarily conserved developmental mechanisms as well as new mechanisms specific to the neocortex that appeared later during evolution and participate in building the mammalian cortex.

## Introduction

Mammalian cerebral cortex comprises the neocortex, the hippocampus, and the olfactory cortex. The olfactory cortex is an evolutionarily conserved paleocortex located in the ventrolateral part of the telencephalon and shares common characteristics with the general cortex of reptiles, which are composed of three thin layers (Aboitiz et al., 2002; Shepherd, 2011; Fournier et al., 2015). This three-layered organization, also called allocortex, is conserved in both the hippocampus (medial pallium derivative) and the olfactory cortex (lateral pallium derivative; Aboitiz et al., 2002). The mammalian olfactory cortex is subdivided into several structures along anteroposterior axis, as follows: the anterior olfactory nucleus, the olfactory tubercle, the piriform cortex, the olfactory amygdala [cortical amygdala and nucleus of the lateral olfactory tract (LOT)], and the lateral entorhinal cortex (lENT; Haberly, 2001; Brunjes et al., 2005; Sanchez-Andrade and Kendrick, 2009). In contrast to the classic sensory pathway where sensory information is first relayed in the thalamus before reaching the neocortex, the olfactory cortex receives direct sensory inputs from the mitral and tufted cells of olfactory bulb (OB) via the LOT, and is thought to play a key role in olfaction (Wilson and Mainen, 2006; Isaacson, 2010; Leinwand and Chalasani, 2011; Bekkers and Suzuki, 2013). The piriform cortex is the most studied structure of the olfactory cortex. It receives inputs from the OB, as well as from the other regions of the olfactory cortex cited above, and sends projections to the anterior olfactory nucleus, the olfactory tubercle, the cortical amygdala (CoA), and the lENT within the olfactory cortex, as well as to the mediodorsal nucleus of the thalamus and to several subdivisions of the prefrontal cortex [including the infralimbic (IL), orbitofrontal, and agranual insular cortices; Johnson et al., 2000; Ekstrand et al., 2001; Meyer et al., 2006; Kerr et al., 2007]. Finally, the piriform cortex sends consequent feedback projections to the OB (Haberly and Price, 1978; Shipley and Adamek, 1984). The piriform cortex has been shown to participate in odor discrimination, association, and learning (Bekkers and Suzuki, 2013); it allows object recognition in a sensory landscape, whose relevant perceptual dimensions are dynamically shaped by sensory experience (Fournier et al., 2015).

While the development of the six-layered neocortex has been extensively described, the development of the olfactory cortex remains poorly understood. Neurons of the olfactory cortex seem to have multiple origins both in the pallial and the subpallial ventricular zones (VZs; Carney et al., 2006; Garcia-Moreno et al., 2008). Moreover, these neurons migrate over long distances and display complex migratory properties before reaching their final destination in the olfactory cortex and organizing into three layers. In this review, we discuss current knowledge on the development and organization of the olfactory cortex and investigate the following questions. How is the olfactory cortex specified during development? Where are the olfactory cortex neurons born? How do they migrate to the olfactory cortex and form the three layers? What is known about neuron diversity within the olfactory cortex, and how do they encode olfactory information? These questions will be addressed keeping the analogy with the neocortex development in order to open evolutionary perspectives on cortical development.

## Arealization and neurogenesis

During the first 10 days of mouse embryonic development, the pallial neuroepithelium proliferates, allowing the exponential generation of progenies through symmetric divisions in the VZ (Noctor et al., 2004). From embryonic day 11 (E11), the first postmitotic neurons are produced by asymmetric divisions, generating both progenies, which stay in the VZ, and neuroblasts, which migrate out of the VZ toward the cortical surface.

### Arealization of the cortex by signaling centers

Based on gene expression, the pallium is subdivided into four major subregions, namely the medial, dorsal, lateral, and ventral pallium; each pallial region is thought to give rise to specific cortical structures, such as the hippocampus, the neocortex, the olfactory cortex, and the amygdala/endopiriform cortex nuclei, respectively (Puelles et al., 2000; Yun et al., 2001). Intrinsic mechanisms based on morphogens and signaling molecules secreted by patterning centers allow the graded expression of transcription factors by cortical progenitors and the development of the different cortical fields (O'Leary et al., 2007; Arai and Pierani, 2014). The following three patterning centers lie at the borders of the telencephalon and participate in the arealization of the cortex: the cortical hem (between cortical and choroidal fields); the commissural plate (at the rostromedial pole of the telencephalon); and the cortical antihem (at the pallial–subpallial boundary, PSB; Mallamaci and Stoykova, 2006). The LIM homeobox protein 2 (LHX2), expressed in a rostrolateral^HIGH^ to caudomedial^LOW^ gradient, suppresses hem and antihem fates, both of which expanded in *Lhx2* mutant mice (Bulchand et al., 2001; Monuki et al., 2001; Nakagawa and O'Leary, 2001; Mangale et al., 2008). The transcription factor FOXG1, expressed in a rostrolateral^HIGH^ to caudomedial^LOW^ gradient, suppresses hem fate and is required for lateral fates, including that of the antihem (Dou et al., 1999; Muzio and Mallamaci, 2005; Hanashima et al., 2007; Shibata et al., 2008). The cortical antihem is of major importance for the specification of the olfactory cortex. In the following section, we will discuss the antihem specification and functions during development (see O'Leary et al., 2007; Subramanian et al., 2009; Montiel and Aboitiz, 2015 for further information about organizing centers and arealization of the cerebral cortex).

### The antihem, major signaling center for the determination of the olfactory cortex

The antihem is located at the PSB, between the ventral pallium and the dorsal lateral ganglionic eminence (dLGE; Yun et al., 2001). The antihem is delineated from adjacent regions through the exclusive expression of the transcription factor *Dbx1* in the ventral pallium VZ, as well as the enriched expression of the secreted Frizzled related gene *sFrp2* (Kim et al., 2001; Yun et al., 2001; Assimacopoulos et al., 2003; Medina et al., 2004). The ventral pallium and the adjacent dLGE both display an enriched expression of *Pax6* transcription factor, which is required for the development of lateral and ventral pallial identities (olfactory cortex and amygdaloid complex; Stoykova et al., 1996; Toresson et al., 2000; Kim et al., 2001; Yun et al., 2001; Hirata et al., 2002; Tole, 2005; Piñon et al., 2008; Cocas et al., 2011).

The position and the specification of the antihem rely on the expression of transcription factors *Pax6*, *Tlx*, and *Gsh2*. In *Pax6* mutant mice, the ventral and the lateral pallium ectopically express subpallial markers, such as *Mash1*, *Gsh2*, and *Dlx2* (Stoykova et al., 1996, 1997, 2000; Toresson et al., 2000; Kim et al., 2001; Yun et al., 2001). *Tlx* mutants display a similar but less dramatic phenotype (Stenman, 2003). In line with *Gsh2* expression in the subpallium, *Gsh2* mutants display pallial gene expression in subpallial domains, such as the dLGE (Toresson et al., 2000; Yun et al., 2001). PAX6 is therefore required for the expression of ventral pallium-specific genes, while GSH2 suppresses their expression in the subpallium (Carney et al., 2009).

The organizer function of the antihem is poorly understood compared with that of the cortical hem in hippocampus development (Subramanian et al., 2009). A prominent palisade of radial glial fibers delineates the PSB. These fibers originate in the corticostriatal junction of the VZ and extend up to the pial surface in the piriform cortex (Molnár and Butler, 2002). In the absence of the antihem or in both *Pax6* and *Tlx* mutants, the radial glial palisade is severely affected at the PSB. Moreover, *Pax6* mutants show a higher number of subpallium-derived interneurons in the cortex, suggesting a role of the radial glial palisade in restricting tangential migration of interneurons during development (Chapouton et al., 1999). In addition, while the antihem expresses Wnt signaling inhibitor *sFrp2*, *Wnt7b* is expressed in the dLGE adjacent to the antihem (Kim et al., 2001; Assimacopoulos et al., 2003). This restriction of the Wnt signaling to the subpallial side of the PSB instructs the position of the radial glial palisade. The antihem further expresses specific molecules, like the epidermal growth factor family members TGF-α, Neuregulin 1 (NRG1) and NRG3, and fibroblast growth factor 7 (Kim et al., 2001; Assimacopoulos et al., 2003). NRG1 has been shown to be essential in the formation and maintenance of the radial glial cells (Anton et al., 1997; Schmid et al., 2003).

### Signaling centers and production of different lineages of pioneer Cajal-Retzius cells

The three organizing centers at the pallial borders are known to be the main origins of Cajal-Retzius (CR) cells (Takiguchi-Hayashi, 2004; Bielle et al., 2005; Yoshida, 2006; Zhao et al., 2006). These early-born pallial cells differentiate between E10.5 and E12.5 in mice and populate the marginal zones of all cortical areas (Smart and Smart, 1977; Wood et al., 1992; Marín-Padilla, 1998). Many CR cells express *Emx1* and *Tbr1* pallial transcription factors (Gorski et al., 2002; Hevner et al., 2003), and Reelin, a secreted glycoprotein that guides radial migration of neocortical neurons (Caviness, 1982; Howell et al., 1997; Alcántara et al., 1998). All CR cells express *Reelin*, but only hem lineage-derived CR cells express the tumor protein 73 (*p73*), while antihem- or septum-derived CR cells specifically express *Dbx1*. This diversity of CR cell lineages may play a role in the development of cytoarchitectonic differences between the neocortex, the olfactory cortex, and the hippocampus (Bielle et al., 2005).

## Migration and layer formation

### Migration and layer formation in the neocortex

Neuron migration in the neocortex has been well studied. From E11, the first neuroblasts accumulate superficially in the neocortical neuroepithelium, forming the preplate beneath the CR cell layer (Marin-Padilla, 1978; Stewart and Pearlman, 1987; Narboux-Nême and Gaspar, 2008; Vitalis and Rossier, 2011). The preplate is a transitory developmental structure in the neocortex of mouse embryos. Axons of preplate neurons are thought to play the role of pioneers for the development of several fiber tracts (Supèr et al., 1998). From E13, new excitatory neurons settle the preplate, dividing it in two layers, the marginal zone at the surface and the subplate near the VZ. Simultaneously, the radial glia develops and its processes reach the pial surface (Rakic, 2003; Borrell and Götz, 2014). These radial glia processes allow the subsequent migration of neuroblasts, which invade the cortical plate in between the marginal zone and the subplate from E13 to E16 (Marin-Padilla, 1978; De Carlos and O’Leary, 1992). Excitatory neurons of cortical layers are then produced in an inside-out manner from the deep to the superficial layers, such that late-born neurons migrate throughout early-born neurons (Molyneaux et al., 2007; Rakic et al., 2009). Interneurons of the neocortex are produced in the ganglionic eminences of the subpallium [medial ganglionic eminence (MGE), LGE, and caudal ganglionic eminence; Anderson et al., 1997] and migrate first tangentially and then radially to settle the cortical layers, starting from E13 (see Fig. 2; Marín and Rubenstein, 2001; Métin et al., 2006; Gelman et al., 2011). MGE-derived interneurons born at different stages settle distinct layers of the neocortex (Miyoshi and Fishell, 2011).

### Early migration to the olfactory cortex

In contrast to the neocortex, the olfactory cortex is located ventrolaterally and therefore does not line the pallial VZ. Complex waves of cell migration from various regions of the VZ toward the olfactory cortex have been described in early mouse development, from E9.75 to E12 (Nomura et al., 2006; Garcia-Moreno et al., 2008). Olfactory neurons generated in the dorsal part of the telencephalon at E9.75 migrate ventrally and align the PSB through Ephrin-A5 repulsive activity, which expression is regulated by PAX6 transcription factor. These ventrally migrating neurons differentiate both in lot cells and olfactory cortex neurons (Nomura et al., 2006).

Lot cells are a specific subpopulation of Reelin^+^/p73^+^ CR cells born from E9.5 to E11.5, which were named after their specific expression of *lot1* (*mGluR1β*; note that lot cells are distinguished from LOT axons by small and capital letters, respectively; Sato et al., 1998; Jiménez et al., 2002; Dixit et al., 2014). Proneural genes *Neurogenin1* and *Neurogenin2* are coexpressed in dorsal and ventral pallial progenitors and required for the differentiation of lot cells (Dixit et al., 2014). These cells are produced in the whole VZ of the pallium from E9.5 to E11.5 and next migrate tangentially and ventrally toward the cortex surface to organize themselves around the presumptive territory of the LOT at E12.5 (Tomioka et al., 2000). The migration and position of lot cells rely on the expression of guidance cues. The neocortex displays dorsoventral gradients of cues, which position the first steps of lot cell migration. The subpallium expresses repulsive cues, which prevent lot cells from invading ventral territories and maintain them superficially at the PSB. Among these cues, Netrin-1 has been shown to locally attract lot cells around the PSB, while Semaphorin 3F is secreted by deep regions of the ventral telencephalon and keeps lot cells at the cerebral surface by its repulsive action (Kawasaki, 2006; Ito et al., 2008). Lot cells act as guideposts for the establishment and positioning of LOT axons, which occur from E12.5 to E13.5 in mice (Squarzoni, 2015). At these early stages, lot cells display long processes and respond to electric stimulation of the OB (Sato et al., 1998; Hirata et al., 2012). Except for lot cells, a general role of CR cells as guideposts for axon development still remains to be elucidated, but CR cells have been involved in the guidance of entorhinal and thalamocortical axons (Ceranik et al., 2000; Del Río et al., 2002; Barber et al., 2015). Recently, de Frutos et al. (2016) discovered a later and unexpected role for lot cells during development: between E13.5 and E15.5, lot cells retract their processes, adopt a rounded-up morphology with small filopodia, and initiate amoeboid-like migration away from the LOT territory to dorsal neocortical regions (de Frutos et al., 2016). This results in a doubled density of CR cells in the neocortex and a 60% reduction of lot CR cell density in the LOT territory. This reallocation of lot CR cells is regulated by the NMDA glutamate receptor and is required for the proper organization of neocortex layer 1 and for layer 2/3 pyramidal cell apical dendrite morphology and spine density (de Frutos et al., 2016). Since the olfactory cortex predates the emergence of the neocortex in evolution, the lot cell reallocation into the neocortex could reflect an evolutionary co-option of these ancestral guideposts. In the neocortex, CR cells are progressively eliminated by apoptosis and disappear by the end of the second postnatal week (Del Río et al., 1996). It is yet unclear whether lot cells remaining in the olfactory cortex are integrated in the olfactory network or whether they are a transient population as well.

**Figure 1.**
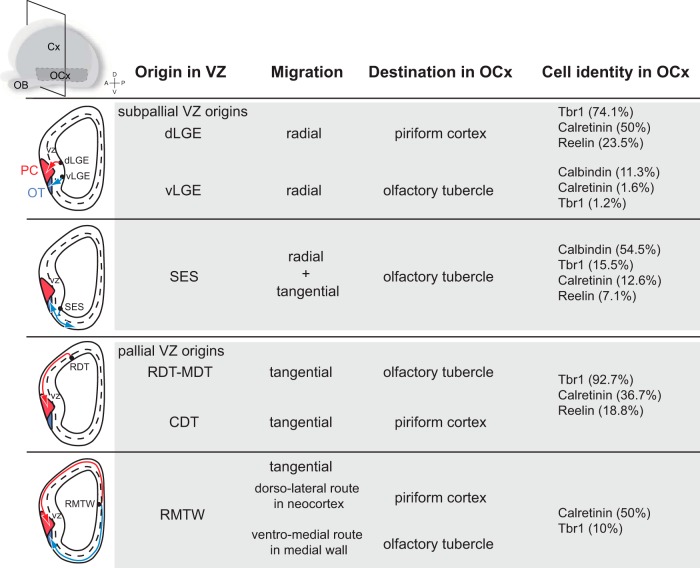
Early migration routes to the olfactory cortex (E10–E12). OCx, Olfactory cortex; PC, piriform cortex; OT, olfactory tubercle; vLGE, ventrolateral ganglionic eminence; SES, septoeminential sulcus; RDT, rostromedial telencephalon; MDT, mediodorsal telencephalon; CDT, caudodorsal telencephalon; RMTW, rostromedial telencephalic wall. In blue, OT migrating neurons; in red, PC migrating neurons. Adapted from Garcia-Moreno et al., 2008, with permission.

Garcia-Moreno et al. (2008) described other migration maps and destination areas of olfactory cortex cells by DiI injections at E10 to E12 followed by *in toto* embryo culture for 1 day (Fig. 1). Neuroblasts are produced in the pallial VZ of the dorsal telencephalon and of the rostromedial telencephalic wall, as well as in the subpallial VZ of the lateral ganglionic eminence and of the septoeminential sulcus. These neuroblasts display tangential or radial migration, with some cells displaying both types of migration sequentially. The authors showed that the cell destination and identity in the olfactory cortex differ depending on their origin in the VZ (Fig. 1). Surprisingly, some neurons from the pallial rostromedial telencephalic wall VZ differentiate in interneurons, while others generated in the dorsal telencephalon differentiate in excitatory neurons. In addition, olfactory cortex neurons from the subpallial dorsal lateral ganglionic eminence VZ differentiate in excitatory neurons, while others generated in the septoeminential sulcus mainly differentiate in interneurons (Garcia-Moreno et al., 2008; Fig. 1). In which olfactory cortex layer these early cells do settle and what are their specific functions in the circuit are not yet elucidated. Further investigations are needed to decipher whether cells coming from different regions in the VZ express specific lineage markers, which could help to identify cell populations and their specific functions within the olfactory cortex.

### Late migration routes to the olfactory cortex

**Figure 2.**
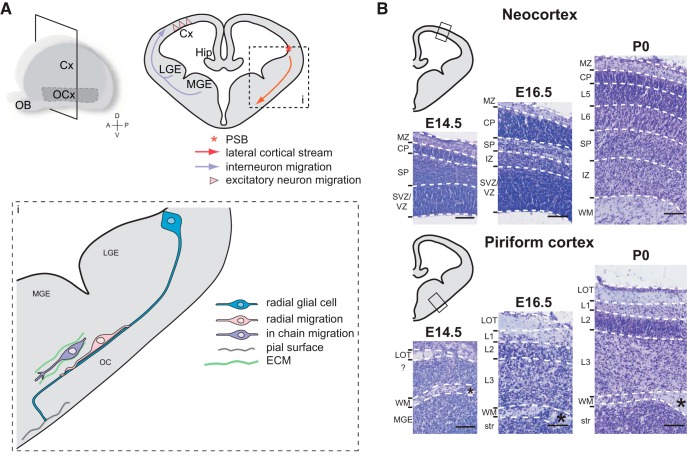
Comparison of developing neocortex and piriform cortex. ***A***, Main migratory routes to the neocortex and to the piriform cortex. i, Lateral cortical stream and modes of migration to the piriform cortex. ***B***, Prenatal development of layers in the neocortex and in the piriform cortex illustrated by cresyl violet stainings. Cx, neocortex; OCx, olfactory cortex; Hip, hippocampus; ECM, extracellular matrix; MZ, marginal zone; CP, cortical plate; SP, subplate; SVZ, subventricular zone; IZ, intermediate zone; WM, white matter; L, layer; str, striatum; *anterior commissure. Scale bars, 200 µm.

The olfactory cortex neurons born in the lateral and ventral pallium at embryonic stages E12 to E15 in rat embryos (corresponding to E11 to E14 in mice) have been initially described to migrate radially toward the surface of the ventrolateral telencephalon (Bayer, 1986; Valverde and Santacana, 1994; De Carlos et al., 1996). The most described migratory route for olfactory cortex neurons is the lateral cortical stream. This migratory route is formed by cells migrating to the piriform cortex, the olfactory tubercle, and the olfactory amygdala. Neuroblasts taking this route are born in the VZ at the PSB and migrate tangentially to the brain surface through ventral regions of the telencephalon, and then radially to settle in the piriform cortex (Fig. 2). In *Pax6* mutant mice (which display an impaired PSB formation), the routing of the lateral cortical stream toward the amygdala and the olfactory cortex is altered (Chapouton et al., 1999; Tole, 2005). In contrast to other known tangential migratory streams described for interneuron migration, the neuroblasts of the lateral cortical stream mainly differentiate into excitatory neurons in the piriform cortex (Corbin et al., 2001). Bai et al. (2008) studied this migratory stream in rat embryos by *in utero* electroporation of a plasmid, allowing monomeric RFP expression by the cells born at E13 in the VZ at the level of the PSB. After 3 days, the cells migrate tangentially toward ventral regions along the lateral cortical stream. These cells settle the piriform cortex after 6 days and mainly differentiate into neuronal transcription factor TBR1^+^ cells (Hevner et al., 2001; Bai et al., 2008). However Carney et al. (2006) showed that the identity of cells migrating along the lateral cortical stream is not homogeneous. The authors described two populations of progenies migrating along the lateral cortical stream in mouse embryos: PAX6^+^ progenies, which derive from the pallium; and DLX2^+^ progenies, which derive from the subpallium. These two populations are generated at precise and distinct temporal windows during development. From E11.5, *Pax6* is highly expressed in the pallium and at the PSB of the VZ. At this stage, some PAX6^+^ cells are visible along the lateral cortical stream and in the piriform cortex. At E15.5, PAX6^+^ cells settle the piriform cortex and the amygdaloid complex. DLX2^+^ cells arose from the PSB about 2 days after PAX6^+^ cells, at E13.5. DLX2^+^ cells are actively migrating from E15.5, and by E18.5 they accumulate in the piriform cortex and the olfactory amygdala. PAX6^+^ cells display a migration along GFAP^+^ radial glial processes found between the PSB of the VZ and the pial surface of the ventrolateral telencephalon at E13.5 and E15.5 (Carney et al., 2006; Fig. 2). Paradoxically, these cells display a tangential migration along radial glial processes. Some DLX2^+^ cells display migration along radial glial processes as well, but “in chain” migration has also been observed in this population (Carney et al., 2006; Fig. 2). The authors hypothesize that PAX6^+^ cells differentiate in excitatory neurons, while DLX2^+^ cells differentiate in inhibitory neurons, since DLX2^+^ cells express the interneuron marker Calbindin (Ghanem et al., 2007).

Zhao et al. (2008) unraveled novel diencephalon-to-telencephalon migrations into the septum, but also into the piriform cortex and the amygdala. By genetically labeling the *Foxb1* diencephalic lineage, the authors identified labeled cells from the caudal hypothalamus, which migrate into ventral levels of the telencephalon through the continuity between the ventral diencephalon and the telencephalon. This migration occurs after the previously described migratory streams: it starts from E15.5 and is substantial at E18.5 in mouse embryos (Zhao et al., 2008). At birth, abundant *Foxb1*-lineage cells migrated from the thalamic region into the globus pallidus, the amygdala, and the piriform cortex. These cells express interneuron markers: most of *Foxb1*-lineage cells in the cortex and in the amygdala express glutamate decarboxylase (Zhao et al., 2008). Some *Foxb1*-lineage cells in the cortex were Calretinin^+^, but none of them expressed Calbindin (Zhao et al., 2008). In rodents, as a rule, cortical interneurons are generated in the ganglionic eminences (Marín and Rubenstein, 2003). The migratory stream uncovered by Zhao et al. (2008) would, therefore, allow a specific pool of interneurons to settle the olfactory cortex during late embryonic stages. Whether these interneurons settle a specific layer in the olfactory cortex or whether they are homogeneously distributed remain an open question, as does their specific function in the neuronal network.

### Layer formation in the olfactory cortex

Reminiscent of the neocortex layer inside-out development, injections of [^3^H]thymidine in E14 to E22 timed-pregnant female rats showed significantly more early-born cells settled in piriform cortex deep layer 3 compared with layer 2 (Bayer, 1986; Valverde and Santacana, 1994). The piriform cortex layer 2 is easily delineable with its high density of pyramidal cell bodies. This layer starts to organize from E16 and is well definable by E18 in mouse embryos (Klingler et al., 2015; Fig. 2). BrdU injections at different mouse development stages and analyses of cell identity at postnatal day 7 showed that cell birth date significantly affects not only the laminar position of cells but also their cellular fate (Sarma et al., 2011). In mice, most layer 2 pyramidal cells are born at E12. E14-born cells are less numerous but still differentiate mainly in pyramidal cells. However, E16-born cells display more heterogeneous identities, as follows: 43% differentiate in pyramidal neurons, 24% differentiate in nonpyramidal neurons (interneurons), and 33% differentiate in non-neuronal cells (astrocytes; Sarma et al., 2011). These results show the conservation of a fundamental developmental chronology in both paleocortices and neocortices, with the sequential generation of pyramidal cells, interneurons, and glia. Labeling of cells generated at precise time points during development could be helpful to identify genetic markers of cell populations in the olfactory cortex. So far, nothing is known about the developmental dynamics of layer 3 neurons.

**Figure 3.**
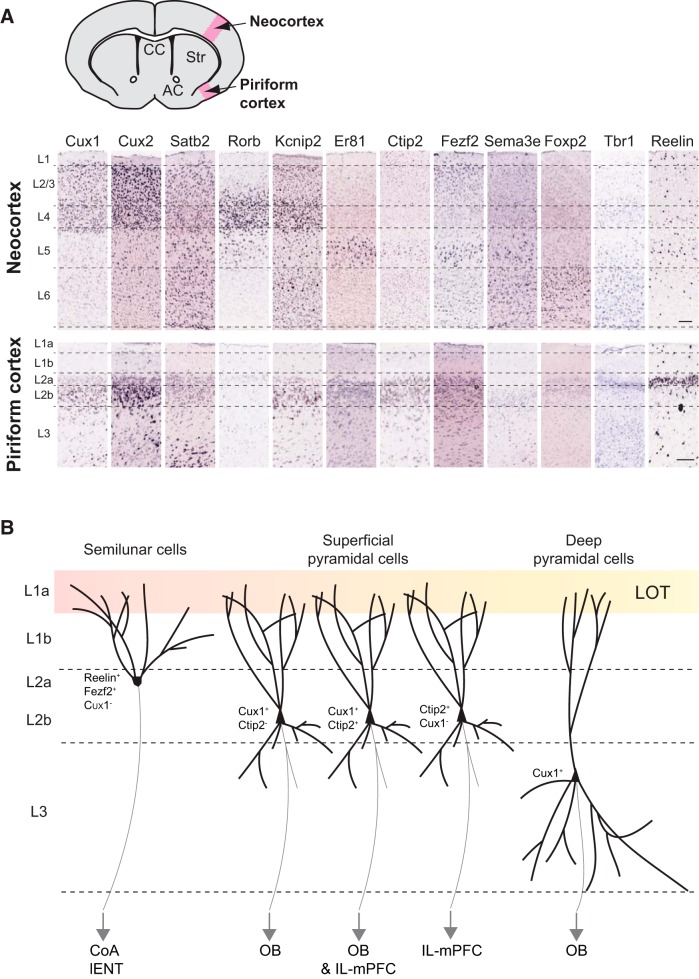
Piriform cortex neuron identities. ***A***, Expression of principal neocortical layer markers in the piriform cortex. *In situ* hybridizations from Allen Brain Atlas database (postnatal day 56). CC, Corpus callosum; AC, anterior commissure; Str, striatum; L, layer. Scale bar, 200 µm. ***B***, Organization, molecular identities, and known targets of projection neurons in the piriform cortex. LOT, lateral olfactory tract; CoA, cortical amygdala; lENT, lateral entorhinal cortex; OB, olfactory bulb; IL-mPFC, infralimbic medial prefrontal cortex.

Axons from OB mitral and tufted cells fasciculate to form the LOT and develop collaterals, which invade the ipsilateral olfactory cortex layer 1 from E15.5 to E17.5 in a caudal to rostral sequence (Fig. 3; Haberly, 1983, 2001; Haberly and Feig, 1983; Hirata and Fujisawa, 1999). Layer 1 can be divided into the following two sublayers: superficial layer 1a formed by the terminals of LOT axons, which make synaptic contacts with the apical dendrites of layer 2/3 pyramidal cells; and layer 1b, which is formed by the apical dendrites of layer 2/3 pyramidal cells (Schwob and Price, 1984; Suzuki and Bekkers, 2011; Fig. 3). In the adult mouse brain, layers 1a and 1b are delineable by MAP-2 and Calretinin immunostainings, which label layer 2/3 pyramidal cell dendrites and LOT axons, respectively (Sarma et al., 2011). At birth, layer 1 is not yet subdivided. Layer 1a starts to be distinguishable from postnatal day 7 with visible costaining of MAP-2 and Calretinin (Sarma et al., 2011). These observations suggest that the synaptic contacts between LOT terminals and layer 2/3 pyramidal cell apical dendrites develop during the first postnatal week. During mouse embryonic development, LOT axons develop collaterals, which first invade the CoA at E15.5, then the piriform cortex 1 day later, followed by the more rostrally located regions of the olfactory cortex (Hirata and Fujisawa, 1999). These collaterals allow one LOT axon to make synaptic contacts with layer 2/3 pyramidal cells of different regions of the olfactory cortex (Ojima et al., 1984). Since LOT axons develop after birth, it is rational to assume that the maturation of the circuit is experience dependent, as it has been well described for the primary somatosensory barrel cortex (for review, see Vitali and Jabaudon, 2014). It would be of interest to investigate the consequences of postnatal odorant stimulus depletion in the organization and maturation of olfactory cortex layer 1 connectivity.

## Cell types and organization of the circuit implicated in odor processing

### Cell types and circuit organization within the piriform cortex

In the piriform cortex, layer 2 pyramidal cells have been initially classified into two populations based on their distinct functions in odor processing: the semilunar and the superficial pyramidal cells (Fig. 3). The semilunar cells are located in the upper part of layer 2 (2a) and display a high spine density in the distal regions of their apical dendrites. These cells receive predominantly afferent excitation from LOT axons, their activity being highly correlated with OB stimulation (Suzuki and Bekkers, 2011). The superficial pyramidal cells are located deeper in layer 2 (2b) and display basal dendrites extending into layer 3 (Fig. 3). They receive weaker afferent inputs from the OB and stronger intracortical excitatory drive through associative fibers, their activity therefore depending on local feedback loops (Haberly and Feig, 1983; Suzuki and Bekkers, 2011; Wiegand et al., 2011; Hagiwara et al., 2012; Fig. 3). Layer 3 is less dense in cell bodies compared with layer 2 and is composed of deep pyramidal cells, which receive minimal afferent inputs from LOT axons, but substantial intracortical excitation (Fig. 3).

To date, molecular identity of the distinct pyramidal neuron within the piriform cortex has been underinvestigated. Most markers defining layers in the neocortex are expressed in the piriform cortex as well (Molyneaux et al., 2007; Fig. 3). Upper layer markers CUX1 and CUX2 (Cubelos et al., 2010) are enriched in piriform cortex layer 2b. Some CUX2^+^ cells are also present in layer 3. Callosal projection neuron marker SATB2 (Alcamo et al., 2008; Leone et al., 2015) is expressed in piriform cortex layers 2 and 3. It would be of interest to know whether SATB2^+^ piriform cortex neurons project to the contralateral hemisphere through the anterior commissure and are therefore the counterparts of SATB2^+^ neocortical callosal projection neurons. Thalamic recipient layer 4 neuron marker RORβ (Jabaudon et al., 2012) shows very weak expression in the piriform cortex. This could be associated with the fact that piriform cortex neurons do not receive sensory input from the thalamus but directly from the OBs. FEZF2 known to be enriched in neocortex layer 5 (Rouaux and Arlotta, 2010; De la Rossa et al., 2013), is enriched in piriform cortex layer 2a (Diodato et al., 2016). Layer 5/6 marker CTIP2 is expressed in piriform cortex layer 2 (Fig. 3). Gene expression comparison between the neocortex and the piriform cortex has been extensively analyzed in the study by Luzzati (2015). He showed that the neocortex layers 2/3 share 42% of enriched genes with the piriform cortex, while deep layers 4 and 6 are less related to the piriform cortex with only 29% of coexpressed genes (Luzzati, 2015). Piriform cortex neurons seem therefore to express common molecular markers enriched in neocortical neurons, but they do not display the same organization within layers. Ramsden et al. (2015) developed a new computational pipeline for high-throughput analysis and comparison of Allen Brain Atlas *in situ* hybridizations at laminar resolution to study gene expression in the medial entorhinal cortex (MEC). As observed for the piriform cortex, very few genes are uniquely expressed in the medial entorhinal cortex. In addition and contrary to the piriform cortex, deep layers of the medial entorhinal cortex are relatively similar to those of the neocortex, while superficial layers are substantially more divergent at a molecular level (Ramsden et al., 2015). The authors proposed that the medial entorhinal cortex is a type of periarchicortex (paleocortex), a transitional structure between the six-layered neocortex and the three-layered archicortex (Ramsden et al., 2015).

A recent study by Diodato et al. (2016) addressed for the first time the molecular identities of piriform cortex projection neurons using laser capture microdissection of piriform cortex layers and RNA deep sequencing to identify genes differentially expressed within piriform cortex layers in combination with retrograde labeling from piriform cortex targets. With these approaches, they showed that layer 2a semilunar cells project to CoA and lENT and express *Reelin* and *Fezf2* (Fig. 3). The expression of *Reelin* by excitatory projection neurons in adult brain is specific to the piriform cortex, since in the neocortex, *Reelin* is mostly expressed by interneurons (Alcántara et al., 1998; Pesold et al., 1998; Ramos-Moreno et al., 2006). These Reelin^+^ piriform cortex semilunar cells resemble the CR cells from the developing brain (Carceller et al., 2016). Carceller et al. (2016) proposed that the secretion of Reelin by upper piriform cortex layer 2 could participate in the maturation of an immature neuron subpopulation expressing polysialylated-neural cell adhesion molecule and Doublecortin located deeper in layer 2 (Nacher et al., 2001; Luzzati et al., 2009; Klempin et al., 2011). Interestingly, Reelin^+^ stellate principal cells have been described in the adult MEC layer 2 (Varga et al., 2010). The MEC layer 2 also comprises pyramidal cells, some of which express Doublecortin late in postnatal development (Ray and Brecht, 2016). A role for Reelin in the late maturation of immature neuron subpopulations can therefore also be considered in the case of the MEC.

Besides, piriform cortex pyramidal neurons sending projections back to OB are located in layer 2b and layer 3, display morphology characteristics of superficial and deep pyramidal cells, and express *Cux1* (Fig. 3). Interestingly, 3D reconstruction of feedback-projecting neurons after rabies virus injection in the granule cell layer of the main OB showed that these cells are more numerous in the anterior than in the posterior piriform cortex and display a nonrandom organization: piriform cortex neurons next to one another project to similar regions of the OB granule cell layer (Padmanabhan et al., 2016). Neurons projecting to IL subdivision of the medial prefrontal cortex (IL-mPFC) are mainly located in layer 2b and express *Ctip2*. Moreover, a fraction of layer 2b neurons express both *Cux1* and *Ctip2* and project to both OB and IL-mPFC (Fig. 3; Diodato et al., 2016). This study points out interesting differences about the molecular organizations of the piriform cortex and the neocortex: while in the neocortex, *Cux1* is expressed by superficial layer neurons, and *Fezf2* by deep layer 5/6 neurons exclusively; this organization is reversed in the piriform cortex, where *Fezf2* is expressed in layer 2a, and *Cux1* in layers 2b and 3. Moreover, *Cux1* and *Ctip2* are expressed in mutually exclusive populations in the neocortex, while a fraction of layer 2b neurons express both markers in the piriform cortex. Interestingly, the authors further showed that in *Reelin*-deficient mice, where cells of distinct layers are intermingled across the piriform cortex, the molecular identities of neurons projecting to the OB (CUX1^+^/FEZF2^−^) compared with neurons projecting to the CoA (CUX1^−^/FEZF2^+^) are conserved, despite their position defect, as described in the neocortex (Ogawa et al., 1995; Hevner et al., 2003; Wagener et al., 2010; Boyle et al., 2011; Diodato et al., 2016).

### Odor processing in the piriform cortex

In mice, each olfactory sensory neuron in the olfactory epithelium expresses only one olfactory receptor gene, and olfactory sensory neurons expressing a common olfactory receptor send convergent projections to two glomeruli in the main OB (Buck and Axel, 1991; Mombaerts et al., 1996; Malnic et al., 1999, 2010). Despite the precise odotopic organization at these levels of the olfactory circuit, individual odorants evoke a response in sparsely and randomly distributed sets of neurons within the piriform cortex (Rennaker et al., 2007; Poo and Isaacson, 2009; Stettler and Axel, 2009; Ghosh et al., 2011; Miyamichi et al., 2011; Sosulski et al., 2011). Using single-cell recordings from head-restrained awake mice, Zhan and Luo (2010) analyzed odor response profiles of individual neurons in the anterior piriform cortex. Upon odorant presentation, 25% of projection neurons were broadly excited and rarely inhibited, 25% showed no excitation and a clear inhibition, and 50% displayed very selective responses in terms of excitation and inhibition (Zhan and Luo, 2010). The apparent random connections from the OB mitral and tufted cell axons to the piriform cortex suggest that the representations of odors in the olfactory cortex are learned by experience. The piriform cortex would detect odors by comparing them with a previously acquired library of odors (Sullivan and Wilson, 2003) and build odor units from the chemicals identified upstream in the olfactory circuit (Johnson et al., 2000). The piriform cortex can therefore be seen as a memory tool optimized in the storage of odor synaptic representations (Barkai et al., 1994).

Olfactory representations within the piriform cortex are strongly shaped by recurrent excitatory and inhibitory intracortical connections (Franks et al., 2011; Poo and Isaacson, 2011). While each piriform cortex pyramidal cell receives only 200 inputs from mitral/tufted OB cells, it receives at least 2000 recurrent excitatory inputs (Davison and Ehlers, 2011; Franks et al., 2011). Contrary to the primary visual and somatosensory cortices, recurrent piriform cortex axons form synapses with the same probability, whatever the distance from the soma (Franks et al., 2011). Local cortical application of baclofen, a GABA_B_ antagonist, abolishes intracortical associational transmission from excitatory neurons without affecting LOT-evoked excitatory responses (Tang and Hasselmo, 1994; Franks and Isaacson, 2005). Using this pharmacologic approach, Poo and Isaacson (2011) showed that the recruitment of intracortical input, rather than OB input, largely determines the strength of odor-evoked excitatory synaptic transmission in the piriform cortex. Using a novel transgenic mouse model allowing the expression of channel rhodopsin (ChR2) in a subset of semilunar cells, Choy et al. (2015) showed that ChR2-expressing semilunar cells avoid targeting neighboring semilunar cells but provide strong monosynaptic associational excitation of superficial and deep pyramidal cells. Piriform cortex interneurons also play an important role in odor processing. They allow increasing discrimination of an odor and prevent nonspecific excitations. Only 10% of neurons will be activated by a given odor (Rennaker et al., 2007; Poo and Isaacson, 2009; Zhan and Luo, 2010). This signal transformation may allow the piriform cortex to perceive a complex mix of odors as an odorant object separated from its pure components. Feedforward inhibition is mediated by layer 1 horizontal and neurogliaform interneurons, which receive direct inputs from the LOT and synapse onto apical dendrites of pyramidal cells, and are thought to play a prominent role in dendritic integration of afferent input from LOT axons in all piriform cortex excitatory cells. Feedback inhibition is mediated by layer 2/3 bitufted regular spiking interneurons, which receive little direct LOT input and synapse onto pyramidal cell bodies and basal dendrites, with deep pyramidal cells receiving the strongest recurrent inhibition (Stokes and Isaacson, 2010; Suzuki and Bekkers, 2010a,b; Taniguchi, 2014; Large et al., 2016). Semilunar cells have been shown to directly activate layer 3 GABAergic interneurons (fast spiking, regular spiking, and neurogliaform), but neither neurogliaform nor horizontal interneurons of layer 1a (Choy et al., 2015). The feedback inhibition would dominate the feedforward inhibition in controlling the activation of piriform pyramidal cells (Franks et al., 2011).

### Synaptic organization of the cortex: from 3 to 6 layers

The cortical type microcircuit consists of a superficial plexiform layer 1, where extrinsic and intrinsic projections meet the apical dendrites of pyramidal neurons located in deeper layers, and is responsible for the generation of recurrent excitation and inhibition (Haberly, 1990). The neocortex shares the basic microcircuits with the three-layered allocortex, but displays a higher number of neurons and layers (Shepherd, 2011; Fournier et al., 2015). The neocortex could then be described as a double allocortex: two couples of pyramidal cell populations, formed by upper layers 2/3 and deeper layers 5/6, are each located below plexiform layers 1 and 4, respectively, carrying extrinsic inputs (Shepherd, 2011; Luzzati, 2015). In contrast to the dorsal cortex of reptiles or to the piriform cortex of mammals, where sensory afferents come from the top, in the neocortex the thalamic afferents ascend from the depth and efferents descend through the same layers. Both inputs and outputs have therefore potential access to all cells in every layer (Shepherd, 2011). Moreover, the generation of superficial and deep layers in the neocortex allows expansion of the combinatorial possibilities for intracortical and subcortical processing (Shepherd, 2011). Neocortex upper layers are evolutionarily the most recent. Interestingly and counterintuitively, piriform cortex neurons express most molecular markers found in these neocortex upper layers. These similarities lead to the hypothesis of an olfactory cortex-based developmental program for the evolution of neocortical layers 2/3 (Luzzati, 2015). The early neocortical column would therefore result of the superposition of the lateral cortex (olfactory cortex) and the dorsal cortex (Luzzati, 2015). Careful studies of piriform cortex cell lineages and neuron molecular identities are needed to understand what specific cell types are found in evolutionary “old” olfactory cortex and neocortex layers.

## Concluding remarks

The olfactory cortex, so-called “simple” cortex in the literature when compared with the six-layered neocortex, is often presented as a general model for cortical sensory processing. However, the molecular identities and the specific functions of cells composing each olfactory cortex layer remain poorly described. The sequential generation of pyramidal cells, interneurons, and glia, as well as the inside-out migration of pyramidal cells seem to be conserved processes in both olfactory cortex and neocortex during development. In the neocortex, excitatory neurons derive from pallial VZ and migrate radially, while inhibitory neurons derive from subpallial VZ and migrate tangentially. This seems not as obvious in the olfactory cortex, where some neurons from the pallium differentiate in inhibitory neurons and some neurons from the subpallium in excitatory neurons (Garcia-Moreno et al., 2008; Fig. 1). The routes of migration toward the olfactory cortex are particularly complicated since olfactory cortex neurons have multiple origins in the VZ and some of them migrate over a long distance to reach their final location. These routes implicate tangential as well as radial migrations. The most studied migratory stream to the olfactory cortex is the lateral cortical stream, which is thought to be the main stream for olfactory cortex excitatory neurons. Some radial glial processes (radial glial palisade) have been identified along this stream and possibly allow the tangential-like migration of excitatory neurons along radial glia (Carney et al., 2006; Fig. 2).

The diversity of their VZ origins implies that the cells composing the olfactory cortex come from different cell lineages and suggests that different cell populations should be definable among olfactory cortex layers. So far, two pyramidal cell populations have mainly been described in the piriform cortex layer 2 based on their morphological and electrophysiological properties (Suzuki and Bekkers, 2011; Fig. 3). However, recent studies started decoding the molecular signatures of projection neurons in respect to their connectivity properties (Diodato et al., 2016; Padmanabhan et al., 2016; Fig. 3). Further investigations will allow understanding whether the different VZ origins and migratory properties of olfactory cortex neurons during development are correlated with their heterogeneous identities and functions in odor processing. Addressing olfactory cortex neuron transcriptional identities throughout development will help to molecularly characterize cell populations with specific properties in the olfactory cortex, in order to compare them with populations identified in the neocortex, in terms of layer localization, connectivity (input and output), and function. The piriform cortex and the dorsal cortex of reptiles (corresponding to visual cortex) both seem to process sensory inputs as high-order cortical areas rather than primary sensory neocortex. Computations performed by high-order cortical areas seem therefore to be ancestral, while computations performed at initial stages of neocortical processing appeared later in evolution, possibly linked to the additions of new layers (Fournier et al., 2015). Unraveling precise neuronal origins and identities in both the neocortex and the olfactory cortex will further elucidate the evolutionarily conserved properties of sensory cortices.

## Synthesis

Reviewing Editor: Esther Stoeckli, University of Zurich Decisions are customarily a result of the Reviewing Editor and the peer reviewers coming together and discussing their recommendations until a consensus is reached. When revisions are invited, a fact-based synthesis statement explaining their decision and outlining what is needed to prepare a revision will be listed below. The following reviewer(s) agreed to reveal their identity: Theofanis Karayannis, Armen Saghatelyan.

First of all, I would like to apologize for the long time it has taken to come to a decision. This is not due to disagreement between the reviewers but unfortunately it has been very challenging to find reviewers during the summer.

As you can see from the individual comments added below, we agree that your review on development and function of the piriform cortex is well written and should be of interest to the readers of ENeuro. However, both reviewers raise important issues that should be addressed. Please carefully evaluate the suggested additional studies that may improve timeliness and coverage of your text.

Specific comments of reviewer 1:

This review is on “The development and organization of the evolutionarily conserved three-layered olfactory cortex”.

The authors start with an introduction about what and where the olfactory cortex is and then continue with 4 sections that cover the organization, neurogenesis and migration of olfactory neurons, followed by the development of the distinct three layers of the olfactory cortex. This is a concise review on mainly the development of the olfactory cortex, but also touches upon its similarities and differences with the more studied neocortex.

From an organizational point of view the authors may want to consider changing the order the information is presented, expanding some, and merging other parts as follows:

- Arealization and neurogenesis

- Migration (early and late) and layer formation

- Cell types and organization of the circuit

At the moment the authors do not give any background on arealization of the cortex by signaling centers and master gene regulators and it may be a good way into the development of the olfactory cortex vs. that of the neocortex.

In addition, whereas the other three current sections have a fair amount of detail the organization section presents some broad knowledge on cell types and has little information on the comparison of the olfactory circuit to the neocortical or that of the ancestral allocortex. Although it is certainly not as much studied as the neocortex, there are a number of reports published not only on the piriform cortex (Zhan and Luo 2010; Franks et al. 2011; Luo 2011; Poo and Isaacson 2011) but on the entorhinal cortex too, especially the medial one which relates to spatial navigation and memory (Cathrin B. Canto et al. Neural Plasticity 2008; Tang Q et al J Neuroscience 2015). Adding some more information on the synaptic organization (see the review by Shepherd GM in Frontiers in Neuroanatomy 2011 ) may also help the reader relate developmental differences with functional ones between the olfactory cortex and neocortex.

The section on “Three olfactory cortex layer(s) formation” is one that I would combine with the “organization of the olfactory cortex”, and have it as the proposed last section “cell types and organization of the circuit”. In the layer section, the authors mention a number of markers, the expression of which is related to that in the neocortex. They may also want to add some data by, for example Ramsden HL et al. Plos Computational Biology 2015 and Ray S and Brecht M Elife 2016, which, although not on piriform, cover some ground on the medial entorhinal cortex.

Also:

Page 1, line 14

Please change to: This comparison the opening of evolutionary...

Page 2, line 27

Since it is a question, please rewrite to: where are the olfactory cortex neurons born?

Page 3, line 5

Change to: ...consists of a superficial

Page 3, line 12

Change to: ...and neurogliaform inhibitory neurons

Page 3, lines 25-26

Please rephrase sentence from:...and receive weaker...

Page 4, line 4

Change to: ...afferent inputs

Page 4, line 8

Change to: ...activity, with deep

Page 4, line 10

The authors may want to expand a little on the concept presented in this paragraph on the spatial to temporal shift, and present some arguments for the evolutionary relationship of the olfactory cortex with the neocortex (reviewed in a paper they also cite, Luzzati F in Frontiers in Neuroscience 2015)

Page 5, line 1

This paragraph presents findings on Cajal-Retzius cells in general, including the ones populating the olfactory cortex. It is not clear what this section adds and whether its content merits its length, since it is not linked to the next paragraph on the lot guidepost cells. Do the authors want to claim that the lot cells are Cajal-Retzius cells? If yes, please consider a better transition between the two paragraphs.

Page 7, line 16

Consider adding some more references. The original observation of where cortical interneurons come from by Anderson S et al Science 1997 and a reference that combines MGE and CGE fate mapping to show when interneurons born at different stages get to the neocortex, such as Miyoshi et al. Cerebral Cortex 2010.

Page 12, line 23

Consider rephrasing this sentence: Further studies...

Page 12, line

Change to: ...find molecular markers and characterize...

Specific comments by reviewer 2:

This review article is interesting, well written and easy to follow. It provides useful information about the development and organization of piriform cortex and discusses it in a critical aspect going beyond the merely descriptive listing of the available information. However, some of the questions raised in the review are based on the assumption that molecular identify of cells in the piriform cortex is not known and deciphering this molecular code will help identifying their connectivity and function. These issues are, however, started to be addressed and recent publications from Diodato et al., 2015; Padmanabhan et al., 2016 provide information about molecular signature and connectivity of these cells. These papers should be cited and manuscript should be revised in the light of this information. Furthermore, while the authors discuss input to different pyramidal cells in the piriform cortex from the olfactory bulb, no information is provided about input from other areas, their output as well as local connections. I believe this information would increase the quality of the work.

1. Pages 2, 4, 7, 11, 12: On several occasions the authors mention that molecular identify of neurons in the piriform cortex is not known and that deciphering this code will help studying their connectivity and role. For example, on the page 2 it is written that "So far, no molecular markers have been described to define the transcriptional identities of principal olfactory cortical neurons...". However, Fleischmann's group has performed laser-capture microdissection, RNA sequencing and tracing experiments to identify molecular factors that mark different neuronal subtypes in the piriform cortex projecting to distinct target areas (Diodata et al., 2015). Furthermore, the authors showed that this molecular code of connectivity is maintained in Reeler mice with disorganized architecture of piriform cortex. These data are not cited in the current manuscript. The authors should consider these data and revised manuscript accordingly.

2. Page 8, line 18: correct "amgladoid"

3. Page 8, lines 25-27: In the chapter "Late migration routes", the authors mention the migration of neuroblasts through the rostral migratory stream (RMS) to the olfactory bulb. This is confusing in the context of this review, since RMS does not provide cells to the piriform cortex.

4. I believe that a chapter on the connectivity of cells in the piriform cortex (both input and output) would improve this review. This is in the subject of this review article about organization of piriform cortex. Several recent articles address this issue using retrograde and anterograde tracing, 3D whole brain reconstructions, transgenic animals and optogenetic stimulation (Diodato et al., 2015; Choy et al., 2015; Padmanabhan et al., 2016).

## References

[B1] Aboitiz F, Montiel J, Morales D, Concha M (2002) Evolutionary divergence of the reptilian and the mammalian brains: considerations on connectivity and development. Brain Res Rev 39:141–153. 12423764 10.1016/s0165-0173(02)00180-7

[B2] Alcamo EA, Chirivella L, Dautzenberg M, Dobreva G, Fariñas I, Grosschedl R, McConnell SK (2008) Satb2 regulates callosal projection neuron identity in the developing cerebral cortex. Neuron 57:364–377. 10.1016/j.neuron.2007.12.012 18255030

[B3] Alcántara S, Ruiz M, D'Arcangelo G, Ezan F, de Lecea L, Curran T, Sotelo C, Soriano E (1998) Regional and cellular patterns of *reelin* mRNA expression in the forebrain of the developing and adult mouse. J Neurosci 18:7779–7799. 9742148 10.1523/JNEUROSCI.18-19-07779.1998 PMC6792998

[B4] Anderson SA, Eisenstat DD, Shi L, Rubenstein JL (1997) Interneuron migration from basal forebrain to neocortex: dependence on Dlx genes. Science 278:474–476. 9334308 10.1126/science.278.5337.474

[B5] Anton ES, Marchionni MA, Lee KF, Rakic P (1997) Role of GGF/neuregulin signaling in interactions between migrating neurons and radial glia in the developing cerebral cortex. Development 124:3501–3510. 9342043 10.1242/dev.124.18.3501

[B6] Arai Y, Pierani A (2014) Development and evolution of cortical fields. Neurosc Res 86:66–76. 10.1016/j.neures.2014.06.005 24983875

[B7] Assimacopoulos S, Grove EA, Ragsdale CW (2003) Identification of a Pax6-dependent epidermal growth factor family signaling source at the lateral edge of the embryonic cerebral cortex. J Neurosci 23:6399–6403. 12878679 10.1523/JNEUROSCI.23-16-06399.2003 PMC6740631

[B8] Bai J, Ramos RL, Paramasivam M, Siddiqi F, Ackman JB, LoTurco JJ (2008) The role of DCX and LIS1 in migration through the lateral cortical stream of developing forebrain. Dev Neurosci 30:144–156. 10.1159/000109859 18075262

[B9] Barber M, Arai Y, Morishita Y, Vigier L, Causeret F, Borello U, Ledonne F, Coppola E, Contremoulins V, Pfrieger FW, Tissir F, Govindan S, Jabaudon D, Proux-Gillardeaux V, Galli T, Pierani A (2015) Migration speed of Cajal-Retzius cells modulated by vesicular trafficking controls the size of higher-order cortical areas. Curr Biol 25:2466–2478. 10.1016/j.cub.2015.08.028 26387718

[B10] Barkai E, Bergman RE, Horwitz G, Hasselmo ME (1994) Modulation of associative memory function in a biophysical simulation of rat piriform cortex. J Neurophysiol 72:659–677. 7527075 10.1152/jn.1994.72.2.659

[B11] Bayer SA (1986) Neurogenesis in the rat primary olfactory cortex. Int J Dev Neurosci 4:251–271. 3455589 10.1016/0736-5748(86)90063-8

[B12] Bekkers JM, Suzuki N (2013) Neurons and circuits for odor processing in the piriform cortex. Trends Neurosci 36:429–438. 10.1016/j.tins.2013.04.005 23648377

[B13] Bielle F, Griveau A, Narboux-Nême N, Vigneau S, Sigrist M, Arber S, Wassef M, Pierani A (2005) Multiple origins of Cajal-Retzius cells at the borders of the developing pallium. Nat Neurosci 8:1002–1012. 10.1038/nn1511 16041369

[B14] Borrell V, Götz M (2014) Role of radial glial cells in cerebral cortex folding. Curr Opin Neurobiol 27:39–46. 10.1016/j.conb.2014.02.007 24632307

[B15] Boyle MP, Bernard A, Thompson CL, Ng L, Boe A, Mortrud M, Hawrylycz MJ, Jones AR, Hevner RF, Lein ES (2011) Cell-type-specific consequences of reelin deficiency in the mouse neocortex, hippocampus, and amygdala. J Comp Neurol 519:2061–2089. 10.1002/cne.22655 21491433

[B16] Brunjes PC, Illig KR, Meyer EA (2005) A field guide to the anterior olfactory nucleus (cortex). Brain Res Reviews 50:305–335. 10.1016/j.brainresrev.2005.08.005 16229895

[B17] Buck L, Axel R (1991) A novel multigene family may encode odorant receptors: a molecular basis for odor recognition. Cell 65:175–187. 1840504 10.1016/0092-8674(91)90418-x

[B18] Bulchand S, Grove EA, Porter FD, Tole S (2001) LIM-homeodomain gene Lhx2 regulates the formation of the cortical hem. Mech Dev 100:165–175. 11165475 10.1016/s0925-4773(00)00515-3

[B19] Carceller H, Rovira-Esteban L, Nacher J, Castrén E, Guirado R (2016) Neurochemical phenotype of Reelin immunoreactive cells in the piriform cortex layer II. Front Cell Neurosci 10:65. 10.3389/fncel.2016.00065 27013976 PMC4785191

[B20] Carney RSE, Alfonso TB, Cohen D, Dai H, Nery S, Stoica B, Slotkin J, Bregman BS, Fishell G, Corbin JG (2006) Cell migration along the lateral cortical stream to the developing basal telencephalic limbic system. J Neurosci 26:11562–11574. 10.1523/JNEUROSCI.3092-06.2006 17093077 PMC6674782

[B21] Carney RSE, Cocas LA, Hirata T, Mansfield K, Corbin JG (2009) Differential regulation of telencephalic pallial-subpallial boundary patterning by Pax6 and Gsh2. Cereb Cortex 19:745–759. 10.1093/cercor/bhn123 18701439 PMC2651477

[B22] Caviness VS (1982) Neocortical histogenesis in normal and reeler mice: a developmental study based upon [3H]thymidine autoradiography. Brain Res 256:293–302. 10.1016/0165-3806(82)90141-9 7104762

[B23] Ceranik K, Zhao S, Frotscher M (2000) Development of the entorhino-hippocampal projection: guidance by Cajal-Retzius cell axons. Ann N Y Acad Sci 911:43–54. 10911866 10.1111/j.1749-6632.2000.tb06718.x

[B24] Chapouton P, Gärtner A, Götz M (1999) The role of Pax6 in restricting cell migration between developing cortex and basal ganglia. Development 126:5569–5579. 10572034 10.1242/dev.126.24.5569

[B25] Choy JMC, Suzuki N, Shima Y, Budisantoso T, Nelson SB, Bekkers JM (2015) Optogenetic mapping of intracortical circuits originating from semilunar cells in the piriform cortex. Cereb Cortex. Advance online publication. Retrieved January 22, 2017. 10.1093/cercor/bhv258 PMC5939214 26503263

[B26] Cocas LA, Georgala PA, Mangin JM, Clegg JM, Kessaris N, Haydar TF, Gallo V, Price DJ, Corbin JG (2011) Pax6 is required at the telencephalic pallial-subpallial boundary for the generation of neuronal diversity in the postnatal limbic system. J Neurosci 31:5313–5324. 10.1523/JNEUROSCI.3867-10.2011 21471366 PMC3086773

[B27] Corbin JG, Nery S, Fishell G (2001) Telencephalic cells take a tangent: non-radial migration in the mammalian forebrain. Nat Neurosci 4[4 Suppl]:1177–1182. 10.1038/nn749 11687827

[B28] Cubelos B, Sebastián-Serrano A, Beccari L, Calcagnotto ME, Cisneros E, Kim S, Dopazo A, Alvarez-Dolado M, Redondo JM, Bovolenta P, Walsh CA, Nieto M (2010) Cux1 and Cux2 regulate dendritic branching, spine morphology, and synapses of the upper layer neurons of the cortex. Neuron 66:523–535. 10.1016/j.neuron.2010.04.038 20510857 PMC2894581

[B29] Davison IG, Ehlers MD (2011) Neural circuit mechanisms for pattern detection and feature combination in olfactory cortex. Neuron 70:82–94. 10.1016/j.neuron.2011.02.047 21482358 PMC3086570

[B30] De Carlos JA, O’Leary DM (1992) Growth and targeting of subplate axons and establishment of major cortical pathways. J Neurosci 12:1194–1211. 1556593 10.1523/JNEUROSCI.12-04-01194.1992 PMC6575791

[B31] De Carlos JA, López-Mascaraque L, Valverde F (1996) Early olfactory fiber projections and cell migration into the rat telencephalon. Int J Dev Neurosci 14:853–866. 9010730 10.1016/s0736-5748(96)00055-x

[B32] de Frutos CA, Bouvier G, Arai Y, Thion MS, Lokmane L, Keita M, Garcia-Dominguez M, Charnay P, Hirata T, Riethmacher D, Grove EA, Tissir F, Casado M, Pierani A, Garel S (2016) Reallocation of olfactory Cajal-Retzius cells shapes neocortex architecture. Neuron 92:435–448. 10.1016/j.neuron.2016.09.020 27693257

[B33] Del Río JA, Heimrich B, Supèr H, Borrell V, Frotscher M, Soriano E (1996) Differential survival of Cajal-Retzius cells in organotypic cultures of hippocampus and neocortex. J Neurosci 16:6896–6907. 8824328 10.1523/JNEUROSCI.16-21-06896.1996 PMC6579265

[B34] del Río JA, Solé M, Borrell V, Martínez A, Soriano E (2002) Involvement of Cajal-Retzius cells in robust and layer-specific regeneration of the entorhino-hippocampal pathways. Eur J Neurosci 15:1881–1890. 12099894 10.1046/j.1460-9568.2002.02027.x

[B35] Diodato A, Ruinart de Brimont M, Yim YS, Derian N, Perrin S, Pouch J, Klatzmann D, Garel S, Choi GB, Fleischmann A (2016) Molecular signatures of neural connectivity in the olfactory cortex. Nat Commun 7:12238 10.1038/ncomms12238 27426965 PMC4960301

[B36] De la Rossa A, Bellone C, Golding B, Vitali I, Moss J, Toni N, Lüscher C, Jabaudon D (2013) In vivo reprogramming of circuit connectivity in postmitotic neocortical neurons. Nat Neurosci 16:193–200. 10.1038/nn.3299 23292682

[B37] Dixit R, Wilkinson G, Cancino GI, Shaker T, Adnani L, Li S, Dennis D, Kurrasch D, Chan JA, Olson EC, Kaplan DR, Zimmer C, Schuurmans C (2014) Neurog1 and Neurog2 control two waves of neuronal differentiation in the piriform cortex. J Neurosci 34:539–553. 10.1523/JNEUROSCI.0614-13.2014 24403153 PMC6608148

[B38] Dou CL, Li S, Lai E (1999) Dual role of brain factor-1 in regulating growth and patterning of the cerebral hemispheres. Cereb Cortex 9:543–550. 10498272 10.1093/cercor/9.6.543

[B39] Ekstrand JJ, Domroese ME, Johnson DMG, Feig SL, Knodel SM, Behan M, Haberly LB (2001) A new subdivision of anterior piriform cortex and associated deep nucleus with novel features of interest for olfaction and epilepsy. J Comp Neurol 434:289–307. 11331530 10.1002/cne.1178

[B40] Fournier J, Müller CM, Laurent G (2015) Looking for the roots of cortical sensory computation in three-layered cortices. Curr Opin Neurobiol 31:119–126. 10.1016/j.conb.2014.09.006 25291080 PMC4898590

[B41] Franks KM, Isaacson JS (2005) Synapse-specific downregulation of NMDA receptors by early experience: a critical period for plasticity of sensory input to olfactory cortex. Neuron 47:101–114. 10.1016/j.neuron.2005.05.024 15996551

[B42] Franks KM, Russo MJ, Sosulski DL, Mulligan AA, Siegelbaum SA, Axel R (2011) Recurrent circuitry dynamically shapes the activation of piriform cortex. Neuron 72:49–56. 10.1016/j.neuron.2011.08.020 21982368 PMC3219421

[B43] Garcia-Moreno F, Lopez-Mascaraque L, de Carlos JA (2008) Early telencephalic migration topographically converging in the olfactory cortex. Cereb Cortex 18:1239–1252. 10.1093/cercor/bhm154 17878174

[B44] Gelman D, Griveau A, Dehorter N, Teissier A, Varela C, Pla R, Pierani A, Marín O (2011) A wide diversity of cortical GABAergic interneurons derives from the embryonic preoptic area. J Neurosci 31:16570–16580. 10.1523/JNEUROSCI.4068-11.2011 22090484 PMC6633309

[B45] Ghanem N, Yu M, Long J, Hatch G, Rubenstein JLR, Ekker M (2007) Distinct cis-regulatory elements from the Dlx1/Dlx2 locus mark different progenitor cell populations in the ganglionic eminences and different subtypes of adult cortical interneurons. J Neurosci 27:5012–5022. 10.1523/JNEUROSCI.4725-06.2007 17494687 PMC4917363

[B46] Ghosh S, Larson SD, Hefzi H, Marnoy Z, Cutforth T, Dokka K, Baldwin KK (2011) Sensory maps in the olfactory cortex defined by long-range viral tracing of single neurons. Nature 472:217–220. 10.1038/nature09945 21451523

[B47] Gorski JA, Talley T, Mengsheng Q, Puelles L, Rubenstein JL, Jones KR (2002) Cortical excitatory neurons and glia, but not GABAergic neurons, are produced in the Emx1-expressing lineage. J Neurosci 22:6309–6314. 12151506 10.1523/JNEUROSCI.22-15-06309.2002 PMC6758181

[B48] Haberly LB (1983) Structure of the piriform cortex of the opossum. I. Description of neuron types with golgi methods. J Comp Neurol 213:163–187. 10.1002/cne.902130205 6841668

[B49] Haberly LB (1990) Comparative aspects of olfactory cortex In: Cerebral cortex (Jones EG, Peters A, eds), pp 137–166. Boston, MA: Springer US.

[B50] Haberly LB (2001) Parallel-distributed processing in olfactory cortex: new insights from morphological and physiological analysis of neuronal circuitry. Chem Senses 26:551–576. 11418502 10.1093/chemse/26.5.551

[B51] Haberly LB, Price JL (1978) Association and commissural fiber systems of the olfactory cortex of the rat. J Comp Neurol 178:711–740. 10.1002/cne.901780408 632378

[B52] Haberly LB, Feig SL (1983) Structure of the piriform cortex of the opossum. II. Fine structure of cell bodies and neuropil. J Comp Neurol 216:69–88. 10.1002/cne.902160107 6863596

[B53] Hagiwara A, Pal SK, Sato TF, Wienisch M, Murthy VN (2012) Optophysiological analysis of associational circuits in the olfactory cortex. Front Neural Circuits 6:18 10.3389/fncir.2012.00018 22529781 PMC3329886

[B54] Hanashima C, Fernandes M, Hebert JM, Fishell G (2007) The role of Foxg1 and dorsal midline signaling in the generation of Cajal-Retzius subtypes. J Neurosci 27:11103–11111. 10.1523/JNEUROSCI.1066-07.2007 17928452 PMC6672859

[B55] Hevner RF, Shi L, Justice N, Hsueh YP, Sheng M, Smiga S, Bulfone A, Goffinet AM, Campagnoni AT, Rubenstein JL (2001) Tbr1 regulates differentiation of the preplate and layer 6. Neuron 353–366. 10.1016/S0896-6273(01)00211-2 11239428

[B56] Hevner RF, Neogi T, Englund C, Daza RAM, Fink A (2003) Cajal–Retzius cells in the mouse: transcription factors, neurotransmitters, and birthdays suggest a pallial origin. Dev Brain Res 141:39–53. 10.1016/S0165-3806(02)00641-7 12644247

[B57] Hirata T, Fujisawa H (1999) Environmental control of collateral branching and target invasion of mitral cell axons during development. J Neurobiol 38:93–104. 10027565

[B58] Hirata T, Nomura T, Takagi Y, Sato Y, Tomioka N, Fujisawa H, Osumi N (2002) Mosaic development of the olfactory cortex with Pax6-dependent and -independent components. Dev Brain Res 136:17–26. 12036513 10.1016/s0165-3806(02)00304-8

[B59] Hirata T, Kumada T, Kawasaki T, Furukawa T, Aiba A, Conquet F, Saga Y, Fukuda A (2012) Guidepost neurons for the lateral olfactory tract: expression of metabotropic glutamate receptor 1 and innervation by glutamatergic olfactory bulb axons. Dev Neurobiol 72:1559–1576. 10.1002/dneu.22030 22539416

[B60] Howell BW, Hawkes R, Soriano E, Cooper JA (1997) Neuronal position in the developing brain is regulated by mouse disabled-1. Nature 389:733–737. 9338785 10.1038/39607

[B61] Isaacson JS (2010) Odor representations in mammalian cortical circuits. Curr Opin Neurobiol 20:328–331. 10.1016/j.conb.2010.02.004 20207132 PMC2896888

[B62] Ito K, Kawasaki T, Takashima S, Matsuda I, Aiba A, Hirata T (2008) Semaphorin 3F confines ventral tangential migration of lateral olfactory tract neurons onto the telencephalon surface. J Neurosci 28:4414–4422. 10.1523/JNEUROSCI.0372-08.2008 18434520 PMC6670952

[B63] Jabaudon D, Shnider SJ, Tischfield DJ, Galazo MJ, Macklis JD (2012) RORβ induces barrel-like neuronal clusters in the developing neocortex. Cereb Cortex 22:996–1006. 10.1093/cercor/bhr182 21799210 PMC3328343

[B64] Jiménez D, López-Mascaraque LM, Valverde F, De Carlos JA (2002) Tangential migration in neocortical development. Dev Biol 244:155–169. 10.1006/dbio.2002.0586 11900465

[B65] Johnson DM, Illig KR, Behan M, Haberly LB (2000) New features of connectivity in piriform cortex visualized by intracellular injection of pyramidal cells suggest that “primary” olfactory cortex functions like “association” cortex in other sensory systems. J Neurosci 20:6974–6982. 10995842 10.1523/JNEUROSCI.20-18-06974.2000 PMC6772836

[B66] Kawasaki T (2006) Netrin 1 regulates ventral tangential migration of guidepost neurons in the lateral olfactory tract. Development 133:845–853. 10.1242/dev.02257 16439477

[B67] Kerr KM, Agster KL, Furtak SC, Burwell RD (2007) Functional neuroanatomy of the parahippocampal region: the lateral and medial entorhinal areas. Hippocampus 17:697–708. 10.1002/hipo.20315 17607757

[B68] Kim AS, Anderson SA, Rubenstein JLR, Lowenstein DH, Pleasure SJ (2001) Pax-6 regulates expression of SFRP-2 and Wnt-7b in the developing CNS. J Neurosci 21:RC132. 11222670 10.1523/JNEUROSCI.21-05-j0002.2001 PMC6762962

[B69] Klempin F, Kronenberg G, Cheung G, Kettenmann H, Kempermann G (2011) Properties of doublecortin-(DCX)-expressing cells in the piriform cortex compared to the neurogenic dentate gyrus of adult mice. PLoS One 6:e25760. 10.1371/journal.pone.0025760 22022443 PMC3192736

[B70] Klingler E, Martin PM, Garcia M, Moreau-Fauvarque C, Falk J, Chareyre F, Giovannini M, Chédotal A, Girault JA, Goutebroze L (2015) The cytoskeleton-associated protein SCHIP1 is involved in axon guidance, and is required for piriform cortex and anterior commissure development. Development 142:2026–2036. 10.1242/dev.119248 25953347

[B71] Large AM, Vogler NW, Mielo S, Oswald A-MM (2016) Balanced feedforward inhibition and dominant recurrent inhibition in olfactory cortex. Proc Natl Acad Sci U S A 113:2276–2281. 10.1073/pnas.1519295113 26858458 PMC4776521

[B72] Leinwand SG, Chalasani SH (2011) Olfactory networks: from sensation to perception. Curr Opin Genet Dev 21:806–811. 10.1016/j.gde.2011.07.006 21889328

[B73] Leone DP, Heavner WE, Ferenczi EA, Dobreva G, Huguenard JR, Grosschedl R, McConnell SK (2015) Satb2 regulates the differentiation of both callosal and subcerebral projection neurons in the developing cerebral cortex. Cereb Cortex 25:3406–3419. 10.1093/cercor/bhu156 25037921 PMC4585495

[B74] Luzzati F (2015) A hypothesis for the evolution of the upper layers of the neocortex through co-option of the olfactory cortex developmental program. Front Neurosci 9:162 10.3389/fnins.2015.00162 26029038 PMC4429232

[B75] Luzzati F, Bonfanti L, Fasolo A, Peretto P (2009) DCX and PSA-NCAM expression identifies a population of neurons preferentially distributed in associative areas of different pallial derivatives and vertebrate species. Cereb Cortex 19:1028–1041. 10.1093/cercor/bhn145 18832334

[B76] Mallamaci A, Stoykova A (2006) Gene networks controlling early cerebral cortex arealization. Eur J Neurosci 23:847–856. 10.1111/j.1460-9568.2006.04634.x 16519650

[B77] Malnic B, Hirono J, Sato T, Buck LB (1999) Combinatorial receptor codes for odors. Cell 96:713–723. 10089886 10.1016/s0092-8674(00)80581-4

[B78] Malnic B, Gonzalez-Kristeller DC, Gutiyama LM (2010) Odorant receptors In: The neurobiology of olfaction (Menini A, ed), pp 181–202. Boca Raton, FL: CRC. 21882436

[B79] Mangale VS, Hirokawa KE, Satyaki PRV, Gokulchandran N, Chikbire S, Subramanian L, Shetty AS, Martynoga B, Paul J, Mai MV, Li Y, Flanagan LA, Tole S, Monuki ES (2008) Lhx2 selector activity specifies cortical identity and suppresses hippocampal organizer fate. Science 319:304–309. 10.1126/science.1151695 18202285 PMC2494603

[B80] Marín O, Rubenstein JL (2001) A long, remarkable journey: tangential migration in the telencephalon. Nat Rev Neurosci 2:780–790. 10.1038/35097509 11715055

[B81] Marin-Padilla M (1978) Dual origin of the mammalian neocortex and evolution of the cortical plate. Anat Embryol 152:109–126. 637312 10.1007/BF00315920

[B82] Marín-Padilla M (1998) Cajal–Retzius cells and the development of the neocortex. Trends Neurosci 21:64–71. 9498301 10.1016/s0166-2236(97)01164-8

[B83] Marín O, Rubenstein JLR (2003) Cell migration in the forebrain. Annu Rev Neurosci 26:441–483. 10.1146/annurev.neuro.26.041002.131058 12626695

[B84] Medina L, Legaz I, González G, De Castro F, Rubenstein JLR, Puelles L (2004) Expression of Dbx1, Neurogenin 2, Semaphorin 5A, Cadherin 8, and Emx1 distinguish ventral and lateral pallial histogenetic divisions in the developing mouse claustroamygdaloid complex. J Comp Neurol 474:504–523. 10.1002/cne.20141 15174069

[B85] Meyer EA, Illig KR, Brunjes PC (2006) Differences in chemo- and cytoarchitectural features within pars principalis of the rat anterior olfactory nucleus suggest functional specialization. J Comp Neurol 498:786–795. 10.1002/cne.21077 16927267 PMC1592518

[B86] Métin C, Baudoin J-P, Rakić S, Parnavelas JG (2006) Cell and molecular mechanisms involved in the migration of cortical interneurons. Eur J Neurosci 23:894–900. 10.1111/j.1460-9568.2006.04630.x 16519654

[B87] Miyamichi K, Amat F, Moussavi F, Wang C, Wickersham I, Wall NR, Taniguchi H, Tasic B, Huang ZJ, He Z, Callaway EM, Horowitz MA, Luo L (2011) Cortical representations of olfactory input by trans-synaptic tracing. Nature 472:191–196. 10.1038/nature09714 21179085 PMC3073090

[B88] Miyoshi G, Fishell G (2011) GABAergic interneuron lineages selectively sort into specific cortical layers during early postnatal development. Cereb Cortex 21:845–852. 10.1093/cercor/bhq155 20732898 PMC3059886

[B89] Molnár Z, Butler AB (2002) The corticostriatal junction: a crucial region for forebrain development and evolution. Bioessays 24:530–541. 10.1002/bies.10100 12111736

[B90] Molyneaux BJ, Arlotta P, Menezes JRL, Macklis JD (2007) Neuronal subtype specification in the cerebral cortex. Nat Rev Neurosci 8:427–437. 10.1038/nrn2151 17514196

[B91] Mombaerts P, Wang F, Dulac C, Chao SK, Nemes A, Mendelsohn M, Edmondson J, Axel R (1996) Visualizing an olfactory sensory map. Cell 87:675–686. 8929536 10.1016/s0092-8674(00)81387-2

[B92] Montiel JF, Aboitiz F (2015) Pallial patterning and the origin of the isocortex. Front Neurosci 9:377 10.3389/fnins.2015.00377 26512233 PMC4604247

[B93] Monuki ES, Porter FD, Walsh CA (2001) Patterning of the dorsal telencephalon and cerebral cortex by a roof Plate-Lhx2 pathway. Neuron 32:591–604. 11719201 10.1016/s0896-6273(01)00504-9

[B94] Muzio L, Mallamaci A (2005) Foxg1 confines Cajal-Retzius neuronogenesis and hippocampal morphogenesis to the dorsomedial pallium. J Neurosci 25:4435–4441. 10.1523/JNEUROSCI.4804-04.2005 15858069 PMC6725101

[B95] Nacher J, Crespo C, McEwen BS (2001) Doublecortin expression in the adult rat telencephalon. Eur J Neurosci 14:629–644. 11556888 10.1046/j.0953-816x.2001.01683.x

[B96] Nakagawa Y, O'Leary DD (2001) Combinatorial expression patterns of LIM-homeodomain and other regulatory genes parcellate developing thalamus. J Neurosci 21:2711–2725. 11306624 10.1523/JNEUROSCI.21-08-02711.2001 PMC6762518

[B97] Narboux-Nême N, Gaspar P (2008) Développement du cortex cérébral: apports récents des études chez la souris et les primates. Èpilepsies 20:220–228.

[B98] Noctor SC, Martínez-Cerdeño V, Ivic L, Kriegstein AR (2004) Cortical neurons arise in symmetric and asymmetric division zones and migrate through specific phases. Nat Neurosci 7:136–144. 10.1038/nn1172 14703572

[B99] Nomura T, Holmberg J, Frisen J, Osumi N (2006) Pax6-dependent boundary defines alignment of migrating olfactory cortex neurons via the repulsive activity of ephrin A5. Development 133:1335–1345. 10.1242/dev.02290 16510508

[B100] O'Leary DDM, Chou S-J, Sahara S (2007) Area patterning of the mammalian cortex. Neuron 56:252–269. 17964244 10.1016/j.neuron.2007.10.010

[B101] Ogawa M, Miyata T, Nakajima K, Yagyu K, Seike M, Ikenaka K, Yamamoto H, Mikoshiba K (1995) The reeler gene-associated antigen on Cajal-Retzius neurons is a crucial molecule for laminar organization of cortical neurons. Neuron 14:899–912. 10.1016/0896-6273(95)90329-1 7748558

[B102] Ojima H, Mori K, Kishi K (1984) The trajectory of mitral cell axons in the rabbit olfactory cortex revealed by intracellular HRP injection. J Comp Neurol 230:77–87. 10.1002/cne.902300107 6096415

[B103] Padmanabhan K, Osakada F, Tarabrina A, Kizer E, Callaway EM, Gage FH, Sejnowski TJ (2016) Diverse representations of olfactory information in centrifugal feedback projections. J Neurosci 36:7535–7545. 10.1523/JNEUROSCI.3358-15.2016 27413162 PMC4945671

[B104] Pesold C, Impagnatiello F, Pisu MG, Uzunov DP, Costa E, Guidotti A, Caruncho HJ (1998) Reelin is preferentially expressed in neurons synthesizing gamma-aminobutyric acid in cortex and hippocampus of adult rats. Proc Natl Acad Sci U S A 95:3221–3226. 10.1073/pnas.95.6.3221 9501244 PMC19723

[B105] Piñon MC, Tuoc TC, Ashery-Padan R, Molnár Z, Stoykova A (2008) Altered molecular regionalization and normal thalamocortical connections in cortex-specific Pax6 knock-out mice. J Neurosci 28:8724–8734. 10.1523/JNEUROSCI.2565-08.2008 18753373 PMC3844775

[B106] Poo C, Isaacson JS (2009) Odor representations in olfactory cortex: “sparse” coding, global inhibition, and oscillations. Neuron 62:850–861. 10.1016/j.neuron.2009.05.022 19555653 PMC2702531

[B107] Poo C, Isaacson JS (2011) A major role for intracortical circuits in the strength and tuning of odor-evoked excitation in olfactory cortex. Neuron 72:41–48. 10.1016/j.neuron.2011.08.015 21982367 PMC3190137

[B108] Puelles L, Kuwana E, Puelles E, Bulfone A, Shimamura K, Keleher J, Smiga S, Rubenstein JLR (2000) Pallial and subpallial derivatives in the embryonic chick and mouse telencephalon, traced by the expression of the genes Dlx‐2, Emx‐1, Nkx‐2.1, Pax‐6, and Tbr‐1. J Comp Neurol 424:409–438. 10.1002/1096-9861(20000828)424:3<409::AID-CNE3>3.0.CO;2-7 10906711

[B109] Rakic P (2003) Developmental and evolutionary adaptations of cortical radial glia. Cereb Cortex 13:541–549. 10.1093/cercor/13.6.541 12764027

[B110] Rakic P, Ayoub AE, Breunig JJ, Dominguez MH (2009) Decision by division: making cortical maps. Trends Neurosci 32:291–301. 10.1016/j.tins.2009.01.007 19380167 PMC3601545

[B111] Ramos-Moreno T, Galazo MJ, Porrero C, Martínez-Cerdeño V, Clascá F (2006) Extracellular matrix molecules and synaptic plasticity: immunomapping of intracellular and secreted Reelin in the adult rat brain. Eur J Neurosci 23:401–422. 10.1111/j.1460-9568.2005.04567.x 16420448

[B112] Ramsden HL, Sürmeli G, McDonagh SG, Nolan MF (2015) Laminar and dorsoventral molecular organization of the medial entorhinal cortex revealed by large-scale anatomical analysis of gene expression. PLoS Comput Biol 11:e1004032 10.1371/journal.pcbi.1004032 25615592 PMC4304787

[B113] Ray S, Brecht M (2016) Structural development and dorsoventral maturation of the medial entorhinal cortex. Elife 5:e13343 10.7554/eLife.13343 27036175 PMC4876644

[B114] Rennaker RL, Chen C-FF, Ruyle AM, Sloan AM, Wilson DA (2007) Spatial and temporal distribution of odorant-evoked activity in the piriform cortex. J Neurosci 27:1534–1542. 10.1523/JNEUROSCI.4072-06.2007 17301162 PMC2291208

[B115] Rouaux C, Arlotta P (2010) Fezf2 directs the differentiation of corticofugal neurons from striatal progenitors in vivo. Nat Neurosci 13:1345–1347. 10.1038/nn.2658 20953195 PMC4207442

[B116] Sanchez-Andrade G, Kendrick KM (2009) The main olfactory system and social learning in mammals. Behav Brain Res 200:323–335. 10.1016/j.bbr.2008.12.021 19150375

[B117] Sarma AA, Richard MB, Greer CA (2011) Developmental dynamics of piriform cortex. Cereb Cortex 21:1231–1245. 10.1093/cercor/bhq199 21041199 PMC3140179

[B118] Sato Y, Hirata T, Ogawa M, Fujisawa H (1998) Requirement for early-generated neurons recognized by monoclonal antibody Lot1 in the formation of lateral olfactory tract. J Neurosci 18:7800–7810. 9742149 10.1523/JNEUROSCI.18-19-07800.1998 PMC6793018

[B119] Schmid RS, McGrath B, Berechid BE, Boyles B, Marchionni M, Sestan N, Anton ES (2003) Neuregulin 1-erbB2 signaling is required for the establishment of radial glia and their transformation into astrocytes in cerebral cortex. Proc Natl Acad Sci U S A 100:4251–4256. 10.1073/pnas.0630496100 12649319 PMC153079

[B120] Schwob JE, Price JL (1984) The development of lamination of afferent fibers to the olfactory cortex in rats, with additional observations in the adult. J Comp Neurol 223:203–222. 10.1002/cne.902230205 6200519

[B121] Shepherd GM (2011) The microcircuit concept applied to cortical evolution: from three-layer to six-layer cortex. Front Neuroanat 5:30 10.3389/fnana.2011.00030 21647397 PMC3102215

[B122] Shibata M, Kurokawa D, Nakao H, Ohmura T, Aizawa S (2008) MicroRNA-9 modulates Cajal-Retzius cell differentiation by suppressing Foxg1 expression in mouse medial pallium. J Neurosci 28:10415–10421. 10.1523/JNEUROSCI.3219-08.2008 18842901 PMC6671033

[B123] Shipley MT, Adamek GD (1984) the connections of the mouse olfactory bulb: a study using orthograde and retrograde transport of wheat germ agglutinin conjugated to horseradish peroxidase. Brain Res Bull 12:669–688. 6206930 10.1016/0361-9230(84)90148-5

[B124] Smart I, Smart M (1977) The location of nuclei of different labelling intensities in autoradiographs of the anterior forebrain of postnatial mice injected with [3H]thymidine on the eleventh and twelfth days post-conception. J Anat 123:512–525. PMC1234548 558181

[B125] Sosulski DL, Lissitsyna Bloom M, Cutforth T, Axel R, Datta SR (2011) Distinct representations of olfactory information in different cortical centres. Nature 472:213–216. 10.1038/nature09868 21451525 PMC3354569

[B126] Squarzoni P (2015) Neuronal and microglial regulators of cortical wiring: usual and novel guideposts. Front Neurosci 9:248 10.3389/fnins.2015.00248 26236185 PMC4505395

[B127] Stenman J (2003) Tlx and Pax6 co-operate genetically to establish the pallio-subpallial boundary in the embryonic mouse telencephalon. Development 130:1113–1122. 12571103 10.1242/dev.00328

[B128] Stettler DD, Axel R (2009) Representations of odor in the piriform cortex. Neuron 63:854–864. 10.1016/j.neuron.2009.09.005 19778513

[B129] Stewart GR, Pearlman AL (1987) Fibronectin-like immunoreactivity in the developing cerebral cortex. J Neurosci 7:3325–3333. 3668630 10.1523/JNEUROSCI.07-10-03325.1987 PMC6569158

[B130] Stokes CCA, Isaacson JS (2010) From dendrite to soma: dynamic routing of inhibition by complementary interneuron microcircuits in olfactory cortex. Neuron 67:452–465. 10.1016/j.neuron.2010.06.029 20696382 PMC2922014

[B131] Stoykova A, Fritsch R, Walther C, Gruss P (1996) Forebrain patterning defects in small eye mutant mice. Development 122:3453–3465. 8951061 10.1242/dev.122.11.3453

[B132] Stoykova A, Götz M, Gruss P, Price J (1997) Pax6-dependent regulation of adhesive patterning, R-cadherin expression and boundary formation in developing forebrain. Development 124:3765–3777. 9367432 10.1242/dev.124.19.3765

[B133] Stoykova A, Treichel D, Hallonet M, Gruss P (2000) Pax6 modulates the dorsoventral patterning of the mammalian telencephalon. J Neurosci 20:8042–8050. 11050125 10.1523/JNEUROSCI.20-21-08042.2000 PMC6772738

[B134] Subramanian L, Remedios R, Shetty A, Tole S (2009) Signals from the edges: the cortical hem and antihem in telencephalic development. Semin Cell Dev Biol 20:712–718. 10.1016/j.semcdb.2009.04.001 19446478 PMC2791850

[B135] Sullivan RM, Wilson DA (2003) Molecular biology of early olfactory memory. Learn Mem 10:1–4. 10.1101/lm.58203 12551958 PMC1913047

[B136] Supèr H, Soriano E, Uylings H (1998) The functions of the preplate in development and evolution of the neocortex and hippocampus. Brain Res Brain Res Rev 27:40–64. 9639671 10.1016/s0165-0173(98)00005-8

[B137] Suzuki N, Bekkers JM (2010a) Inhibitory neurons in the anterior piriform cortex of the mouse: classification using molecular markers. J Comp Neurol 518:1670–1687. 20235162 10.1002/cne.22295

[B138] Suzuki N, Bekkers JM (2010b) Distinctive classes of GABAergic interneurons provide layer-specific phasic inhibition in the anterior piriform cortex. Cereb Cortex 20:2971–2984. 20457693 10.1093/cercor/bhq046 PMC2978245

[B139] Suzuki N, Bekkers JM (2011) Two layers of synaptic processing by principal neurons in piriform cortex. J Neurosci 31:2156–2166. 10.1523/JNEUROSCI.5430-10.2011 21307252 PMC6633060

[B140] Takiguchi-Hayashi K (2004) Generation of Reelin-positive marginal zone cells from the caudomedial wall of telencephalic vesicles. J Neurosci 24:2286–2295. 10.1523/JNEUROSCI.4671-03.2004 14999079 PMC6730420

[B141] Tang AC, Hasselmo ME (1994) Selective suppression of intrinsic but not afferent fiber synaptic transmission by baclofen in the piriform (olfactory) cortex. Brain Res 659:75–81. 10.1016/0006-8993(94)90865-6 7820683

[B142] Taniguchi H (2014) Genetic dissection of GABAergic neural circuits in mouse neocortex. Front Cell Neurosci 8:8. 24478631 10.3389/fncel.2014.00008 PMC3902216

[B143] Tole S (2005) Selective requirement of Pax6, but not Emx2, in the specification and development of several nuclei of the amygdaloid complex. J Neurosci 25:2753–2760. 10.1523/JNEUROSCI.3014-04.2005 15758185 PMC6725176

[B144] Tomioka N, Osumi N, Sato Y, Inoue T, Nakamura S, Fujisawa H, Hirata T (2000) Neocortical origin and tangential migration of guidepost neurons in the lateral olfactory tract. J Neurosci 20:5802–5812. 10908621 10.1523/JNEUROSCI.20-15-05802.2000 PMC6772553

[B145] Toresson H, Potter SS, Campbell K (2000) Genetic control of dorsal-ventral identity in the telencephalon: opposing roles for Pax6 and Gsh2. Development 127:4361–4371. 11003836 10.1242/dev.127.20.4361

[B146] Valverde F, Santacana M (1994) Development and early postnatal maturation of the rat primary olfactory cortex. Dev Brain Res 80:96–114. 10.1016/0165-3806(94)90093-0 7955365

[B147] Varga C, Lee SY, Soltesz I (2010) Target-selective GABAergic control of entorhinal cortex output. Nat Neurosci 13:822–824. 10.1038/nn.2570 20512133 PMC3139425

[B148] Vitali I, Jabaudon D (2014) Synaptic biology of barrel cortex circuit assembly. Semin Cell Dev Biol 35:156–164. 10.1016/j.semcdb.2014.07.009 25080022

[B149] Vitalis T, Rossier J (2011) New insights into cortical interneurons development and classification: contribution of developmental studies. Dev Neurobiol 71:34–44. 10.1002/dneu.20810 21154908

[B150] Wagener RJ, Dávid C, Zhao S, Haas CA, Staiger JF (2010) The somatosensory cortex of reeler mutant mice shows absent layering but intact formation and behavioral activation of columnar somatotopic maps. J Neurosci 30:15700–15709. 10.1523/JNEUROSCI.3707-10.2010 21084626 PMC6633666

[B151] Wiegand HF, Beed P, Bendels MHK, Leibold C, Schmitz D, Johenning FW (2011) Complementary sensory and associative microcircuitry in primary olfactory cortex. J Neurosci 31:12149–12158. 10.1523/JNEUROSCI.0285-11.2011 21865457 PMC6623216

[B152] Wilson RI, Mainen ZF (2006) Early events in olfactory processing. Annu Rev Neurosci 29:163–201. 16776583 10.1146/annurev.neuro.29.051605.112950

[B153] Wood JG, Martin S, Price DJ (1992) Evidence that the earliest generated cells of the murine cerebral cortex form a transient population in the subplate and marginal zone. Dev Brain Res 66:137–140. 10.1016/0165-3806(92)90150-U 1600628

[B154] Yoshida M (2006) Massive loss of Cajal-Retzius cells does not disrupt neocortical layer order. Development 133:537–545. 10.1242/dev.02209 16410414

[B155] Yun K, Potter S, Rubenstein JL (2001) Gsh2 and Pax6 play complementary roles in dorsoventral patterning of the mammalian telencephalon. Development 128:193–205. 11124115 10.1242/dev.128.2.193

[B156] Zhan C, Luo M (2010) Diverse patterns of odor representation by neurons in the anterior piriform cortex of awake mice. J Neurosci 30:16662–16672. 10.1523/JNEUROSCI.4400-10.2010 21148005 PMC6634870

[B157] Zhao C, Guan W, Pleasure SJ (2006) A transgenic marker mouse line labels Cajal–Retzius cells from the cortical hem and thalamocortical axons. Brain Res 1077:48–53. 10.1016/j.brainres.2006.01.042 16490185

[B158] Zhao T, Szabó N, Ma J, Luo L, Zhou X, Alvarez-Bolado G (2008) Genetic mapping of Foxb1-cell lineage shows migration from caudal diencephalon to telencephalon and lateral hypothalamus. Eur J Neurosci 28:1941–1955. 10.1111/j.1460-9568.2008.06503.x 19046377 PMC2777254

